# Supplementary material for: Mapping patterns of thought onto brain activity during movie-watching
Source: eLife. 2025 Jan 10;13:RP97731. doi: 10.7554/eLife.97731 (PMC11723579; doi:10.7554/eLife.97731)
Supplement: Supplementary file 1. — (a) Multi-dimensional Experience sampling (mDES). (b) Percent variance explained by principal components by movie (c) Linear Mixed Models of Variance in Thoughts across Movies. (d) Movie Comprehension Questions. (e) Linear Mixed Models of Comprehension model. (f) FSL FEAT Query Parameter Estimates. (g) Grand average of Gradient score by movie. (h) Functional Connectivity Cluster Analysis (FLAME). (i) Neurosynth Decoder Analysis. (j) Linear Mixed Models of Gradients 1–5 Fixed Effects for each Thought Pattern [file elife-97731-supp1.docx]

**Supplementary File 1a**

*Multi-dimensional Experience sampling (mDES)*

| Questions | Label | Low | High |
| --- | --- | --- | --- |
| My thoughts were focused on an external task or activity: | Focus | Not at all | Completely |
| My thoughts involved future events: | Future | Not at all | Completely |
| My thoughts involved past events: | Past | Not at all | Completely |
| My thoughts involved myself: | Self | Not at all | Completely |
| My thoughts involved other people: | Other | Not at all | Completely |
| The emotion of my thoughts was: | Emotion | Negative | Positive |
| My thoughts involved images | Images | Not at all | Completely |
| My thoughts were detailed and specific: | Detailed | Not at all | Completely |
| My thoughts were: | Deliberate | Spontaneous | Deliberate |
| I was thinking about solutions to problems (or goals): | Problem | Not at all | Completely |
| My thoughts were intrusive: | Intrusive | Not at all | Completely |
| My thoughts contained information I already knew (e.g., knowledge or memories): | Knowledge | Not at all | Completely |
| I was absorbed in the contents of my thoughts | Absorption | Not at all | Completely |
| My thoughts were distracting me from what I am doing | Distracting | Not at all | Completely |
| My thoughts involved words | Words | Not at all | Completely |
| My thoughts involved sounds | Sounds | Not at all | Completely |

**Supplementary File 1b**

| *Percent variance explained by principal components by movie* |
| --- |

| **Movie** | PC1 | PC2 | PC3 | PC4 | Total |
| --- | --- | --- | --- | --- | --- |
| ***Citizenfour*** | 14.01% | 14.97% | 12.85% | 8.51% | 50.34% |
| ***Little Miss Sunshine*** | 14.93% | 13.02% | 13.67% | 9.77% | 51.43% |
| ***500 Days of Summer*** | 18.22% | 11.26% | 12.28% | 9.61% | 51.38% |
| **Combined Dataset** | 26.07% | 10.45% | 7.73% | 6.77% | 51.03% |

**Supplementary File 1c**

*Linear Mixed Models of Variance in Thoughts across Movies*

| Model 1 |  |  |  |  |  |  |  |
| --- | --- | --- | --- | --- | --- | --- | --- |
|  |  | Sum Sq. | Mean Sq. | Num DF | Den DF | F value | p-value |
|  | Movie | 16.83 | 8.41 | 2 | 2015.30 | 5.41 | 0.005 |
| Model 1 lsmeans | |  |  |  |  |  |  |
|  |  | Contrasts | lsmean | SE | df | t-value | p-value |
|  |  | c4 - lms | -0.02 | .10 | 159 | 1.31 | .392 |
|  |  | c4 - summer | -0.11 | .10 | 159 | -1.97 | .121 |
|  |  | lms - summer | 0.11 | .10 | 160 | -3.27 | .003 |
|  |  |  |  |  |  |  |  |
| Model 2 |  |  |  |  |  |  |  |
|  |  | Sum Sq. | Mean Sq. | Num DF | Den DF | F value | p-value |
|  | Movie | 186.92 | 93.46 | 2 | 2015.30 | 77.84 | < .001 |
| Model 2 lsmeans | |  |  |  |  |  |  |
|  |  | Contrasts | lsmean | SE | df | t-value | p-value |
|  |  | c4 - lms | 0.56 | .06 | 2015 | 9.66 | < .001 |
|  |  | c4 - summer | 0.68 | .06 | 2016 | 11.66 | < .001 |
|  |  | lms - summer | 0.12 | .06 | 2016 | 2.03 | .106 |
|  |  |  |  |  |  |  |  |
| Model 3 |  |  |  |  |  |  |  |
|  |  | Sum Sq. | Mean Sq. | Num DF | Den DF | F value | p-value |
|  | Movie | 34.29 | 17.15 | 2 | 2015.40 | 12.90 | < .001 |
| Model 3 lsmeans | |  |  |  |  |  |  |
|  |  | Contrasts | lsmean | SE | df | t-value | p-value |
|  |  | c4 - lms | 0.30 | .06 | 2015 | 5.14 | < .001 |
|  |  | c4 - summer | 0.21 | .06 | 2016 | 2.58 | .001 |
|  |  | lms - summer | -0.09 | .06 | 2016 | -1.54 | .271 |
|  |  |  |  |  |  |  |  |
| Model 4 |  |  |  |  |  |  |  |
|  |  | Sum Sq. | Mean Sq. | Num DF | Den DF | F value | p-value |
|  | Movie | 149.90 | 74.95 | 2 | 2015.70 | 82.69 | < .001 |
| Model 4 lsmeans | |  |  |  |  |  |  |
|  |  | Contrasts | lsmean | SE | df | t-value | p-value |
|  |  | c4 - lms | -0.31 | .05 | 2015 | -6.07 | < .001 |
|  |  | c4 - summer | -0.65 | .05 | 2016 | -12.85 | < .001 |
|  |  | lms - summer | -0.34 | .05 | 2016 | -6.81 | < .001 |

**Supplementary File 1d**

*Movie Comprehension Questions*

| Movie | Question | Answer |
| --- | --- | --- |
| *Citizenfour* | What animal is seen on screen in the first scene? | Dog |
| *Citizenfour* | What president is being discussed in the phone call with David? | Barack Obama |
| *Citizenfour* | After 4 years in the military, who directly entered the NSA with 37 years of combined service? | William |
| *Citizenfour* | According to the movie, what historical event triggered the government to begin spying on citizens? | 11-Sep |
| *Little Miss Sunshine* | What breakfast item did Olive order a la mode? | Waffles |
| *Little Miss Sunshine* | What colour is the Volkswagen Transporter that the family drives? | Yellow |
| *Little Miss Sunshine* | How does the family get into their car after taking it to the repair shop? | Pushing it |
| *Little Miss Sunshine* | What type of language does Richard claim is “the refuge of losers”? | Sarcasm |
| *500 Days of Summer* | What event are both Summer and Tom attending? | Wedding |
| *500 Days of Summer* | What piece of jewelry is Summer showing her friend when Tom abruptly leaves her party? | Engagement ring |
| *500 Days of Summer* | On day 440, what time does Tom’s alarm clock go off? | 7:00 AM |
| *500 Days of Summer* | What book is Tom reading on the train, which he also gifts to Summer at her party? | Architecture of happiness |

**Supplementary File 1e**

*Linear Mixed Models of Comprehension model*

|  | Sum Sq. | Mean Sq. | Num Df | Den Df | F-value | p-value |
| --- | --- | --- | --- | --- | --- | --- |
| Movie | 47.32 | 23.66 | 2 | 254.12 | 49.33 | < .001 |
| PCA_1 | 0.64 | 0.64 | 1 | 338.84 | 1.32 | .376 |
| PCA_2 | 4.45 | 4.45 | 1 | 324.31 | 8.27 | .011 |
| PCA_3 | 0.01 | 0.01 | 1 | 332.52 | 0.03 | .865 |
| PCA_4 | 3.98 | 3.98 | 1 | 341.44 | 8.30 | .013 |
| Movie*PCA_1 | 4.28 | 2.14 | 2 | 268.96 | 3.36 | .013 |
| Movie*PCA_2 | 1.18 | 0.89 | 2 | 259.95 | 1.85 | .286 |
| Movie*PCA_3 | 1.01 | 0.51 | 2 | 264.49 | 1.05 | .450 |
| Movie*PCA_4 | 0.32 | 0.16 | 2 | 269.18 | 0.33 | .810 |

*Pairwise Comparisons for significant main effect of movie*

| Contrasts | Estimate | SE | Df | t-value | p-value |
| --- | --- | --- | --- | --- | --- |
| c4 - lms | -0.93 | .10 | 249 | -9.16 | < .001 |
| c4 - summer | -0.91 | .11 | 273 | -8.33 | < .001 |
| lms - summer | 0.02 | .10 | 242 | 0.18 | .982 |

*Note.* The abbreviation ‘c4’ refers to *Citizenfour*, ‘lms’ refers to *Little Miss Sunshine*, and ‘summer’ refers to *500 Days of Summer*.

**Supplementary File 1f**

*FSL FEAT Query Parameter Estimates*

| **All Movies** |  |  |  |
| --- | --- | --- | --- |
|  | Mean | LCI | UCI |
| Episodic Knowledge | 0.62 | 0.27 | 0.97 |
| Intrusive Distraction | -0.78 | -1.37 | -0.20 |
| Verbal Detail | -1.64 | -2.11 | -1.18 |
| Sensory Engagement | 1.26 | 0.81 | 1.71 |
|  |  |  |  |
| ***500 Days of Summer*** |  |  |  |
|  | Mean | LCI | UCI |
| Episodic Knowledge | -0.23 | -0.58 | 0.12 |
| Intrusive Distraction | -2.34 | -2.89 | -1.80 |
| Verbal Detail | -0.87 | -1.25 | -0.48 |
| Sensory Engagement | 0.18 | -0.02 | 0.39 |
|  |  |  |  |
| ***Citizenfour*** |  |  |  |
|  | Mean | LCI | UCI |
| Episodic Knowledge | 1.49 | 1.06 | 1.91 |
| Intrusive Distraction | 0.26 | -0.26 | 0.78 |
| Verbal Detail | -2.98 | -3.64 | -2.31 |
| Sensory Engagement | 2.77 | 2.27 | 3.26 |
|  |  |  |  |
| ***Little Miss Sunshine*** |  |  |  |
|  | Mean | LCI | UCI |
| Episodic Knowledge | 0.88 | -0.15 | 1.90 |
| Intrusive Distraction | 1.29 | -0.75 | 3.33 |
| Verbal Detail | -0.25 | -1.00 | 0.51 |
| Sensory Engagement | 0.29 | -0.60 | 1.19 |

**Supplementary File 1g**

*Grand average of Gradient score by movie*

|  | **Gradient 1 mean** | **Gradient 2 mean** | **Gradient 3 mean** | **Gradient 4 mean** | **Gradient 5 mean** |
| --- | --- | --- | --- | --- | --- |
| *500 Days of Summer* | -0.00100 | 0.00264 | 0.00201 | 0.00134 | -0.00112 |
| *Citizenfour* | 0.00301 | -0.00191 | 0.00097 | 0.00160 | 0.00000 |
| *Little Miss Sunshine* | 0.00332 | 0.00138 | -0.00099 | 0.00057 | 0.00130 |

**Supplementary File 1h**

*Functional Connectivity Cluster Analysis (FLAME)*

| Episodic Knowledge Functional Connectivity | | | | |  |  |  |  |  |  |
| --- | --- | --- | --- | --- | --- | --- | --- | --- | --- | --- |
| Cluster Index | Voxels | *p* | log *p* | Z-MAX | Z-MAX X (mm) | Z-MAX Y (mm) | Z-MAX Z (mm) | Z-COG X (mm) | Z-COG Y (mm) | Z-COG Z (mm) |
| 3 | 286 | 3.04E-06 | 5.52 | 4.47 | -8 | -100 | 14 | -8.53 | -97.3 | 13.4 |
| 2 | 184 | 0.000197 | 3.71 | 3.93 | 6 | -92 | 14 | 11.3 | -93.5 | 17.5 |
| 1 | 101 | 0.0109 | 1.96 | 3.84 | -58 | -16 | 6 | -58.2 | -15.2 | 6.22 |
|  |  |  |  |  |  |  |  |  |  |  |
| Intrusive Distraction Functional Connectivity | | | | |  |  |  |  |  |  |
| Cluster Index | Voxels | *p* | log *p* | Z-MAX | Z-MAX X (mm) | Z-MAX Y (mm) | Z-MAX Z (mm) | Z-COG X (mm) | Z-COG Y (mm) | Z-COG Z (mm) |
| 4 | 547 | 1.11E-09 | 8.95 | 4.8 | -34 | -58 | 50 | -36 | -52.6 | 46.4 |
| 3 | 408 | 5.96E-08 | 7.22 | 4.3 | -46 | 8 | 26 | -45.9 | 6.9 | 29.3 |
| 2 | 289 | 4.89E-06 | 5.31 | 4.04 | 42 | -46 | 44 | 38.4 | -51.3 | 45.6 |
| 1 | 130 | 0.00338 | 2.47 | 4.08 | 46 | 48 | -4 | 45 | 48.7 | 1.19 |
|  |  |  |  |  |  |  |  |  |  |  |
| Verbal Detail Functional Connectivity | | | |  |  |  |  |  |  |  |
| Cluster Index | Voxels | *p* | log *p* | Z-MAX | Z-MAX X (mm) | Z-MAX Y (mm) | Z-MAX Z (mm) | Z-COG X (mm) | Z-COG Y (mm) | Z-COG Z (mm) |
| 4 | 1895 | 7.05E-24 | 23.2 | 5.24 | -60 | -16 | 6 | -56.3 | -23.3 | 4.47 |
| 3 | 1010 | 2.10E-15 | 14.7 | 4.74 | 58 | -8 | 4 | 58.3 | -18.4 | 3.45 |
| 2 | 105 | 0.00967 | 2.01 | 3.82 | 12 | -88 | 10 | 10.6 | -86.9 | 11 |
| 1 | 75 | 0.05 | 1.3 | 3.74 | -52 | -76 | 6 | -50.3 | -76.6 | 4.32 |
|  |  |  |  |  |  |  |  |  |  |  |
| Sensory Engagement Functional Connectivity | | | | |  |  |  |  |  |  |
| Cluster  Index | Voxels | *p* | log *p* | Z-MAX | Z-MAX X (mm) | Z-MAX Y (mm) | Z-MAX Z (mm) | Z-COG X (mm) | Z-COG Y (mm) | Z-COG Z (mm) |
| 5 | 4566 | 7.43E-44 | 43.1 | 5.23 | 6 | -92 | 16 | 9.59 | -80.2 | 1.36 |
| 4 | 340 | 4.77E-07 | 6.32 | 4.22 | -46 | -80 | 2 | -46.4 | -76.7 | 5.65 |
| 3 | 330 | 6.56E-07 | 6.18 | 4.46 | 62 | -8 | 4 | 61.3 | -10.7 | 7.1 |
| 2 | 290 | 2.92E-06 | 5.53 | 4.59 | -64 | -12 | 6 | -61.1 | -13.4 | 5.4 |
| 1 | 156 | 0.000754 | 3.12 | 3.68 | -8 | -60 | 66 | -6.45 | -56.5 | 58.4 |

**Supplementary File 1i**

*Neurosynth Decoder Analysis*

| Episodic Knowledge |  | Intrusive Distraction |  | Verbal Detail |  | Sensory Engagement |  |
| --- | --- | --- | --- | --- | --- | --- | --- |
| association term |  | association term |  | association term |  | association term |  |
|  | *r* |  | *r* |  | *r* |  | *r* |
| cuneus | 0.17 | working | 0.33 | auditory cortex | 0.65 | v1 | 0.40 |
| v1 | 0.17 | working memory | 0.33 | sounds | 0.65 | visual | 0.35 |
| primary visual | 0.13 | intraparietal | 0.31 | auditory | 0.64 | visual cortex | 0.34 |
| sighted | 0.11 | intraparietal sulcus | 0.30 | primary auditory | 0.62 | primary visual | 0.32 |
| pointing | 0.08 | parietal cortex | 0.30 | heschl | 0.61 | occipital | 0.27 |
| visual | 0.08 | tasks | 0.29 | sound | 0.61 | extrastriate | 0.25 |
| occipital | 0.07 | task | 0.27 | heschl gyrus | 0.60 | early visual | 0.25 |
| videos | 0.07 | posterior parietal | 0.27 | speech | 0.60 | mt | 0.24 |
| occipital gyrus | 0.06 | sulcus ips | 0.25 | listening | 0.59 | sighted | 0.23 |
| visual stream | 0.05 | parietal | 0.25 | pitch | 0.59 | motion | 0.21 |
| prefrontal | -0.02 | resting state | -0.18 | prefrontal | -0.16 | prefrontal | -0.08 |
| cerebellum | -0.02 | resting | -0.18 | prefrontal cortex | -0.13 | cingulate | -0.08 |
| premotor | -0.02 | amygdala | -0.11 | cingulate | -0.06 | prefrontal cortex | -0.07 |
| memory | -0.02 | controls | -0.11 | medial | -0.05 | cerebellum | -0.05 |
| movements | -0.02 | emotional | -0.11 | parietal | -0.04 | motor | -0.05 |
| motor | -0.02 | disorder | -0.10 | memory | -0.04 | sensorimotor | -0.05 |
| primary motor | -0.02 | orbitofrontal | -0.08 | cerebellum | -0.04 | primary motor | -0.05 |
| movement | -0.02 | stimulation | -0.06 | cerebellar | -0.04 | memory | -0.05 |
| parietal | -0.02 | cingulate | -0.06 | disorder | -0.04 | movement | -0.05 |
| pain | -0.02 | disorders | -0.05 | task | -0.03 | motor cortex | -0.05 |

**Supplementary File 1j**

*Linear Mixed Models of Gradients 1-5 Fixed Effects for each Thought Pattern*

| **DV: Episodic Knowledge** | | |  |  |  |
| --- | --- | --- | --- | --- | --- |
|  | Estimate | Std. Error | Df | t-value | p-value |
| (Intercept) | 0.01 | 0.09 | 118.6 | 0.07 | .940 |
| gradient1 | -0.01 | 0.03 | 2043 | -0.26 | .796 |
| gradient2 | -0.04 | 0.04 | 2043 | -0.94 | .579 |
| gradient3 | -0.03 | 0.04 | 2042 | -0.69 | .613 |
| gradient4 | 0.18 | 0.06 | 2046 | 3.02 | .013 |
| gradient5 | -0.18 | 0.08 | 2045 | -2.24 | .063 |
|  |  |  |  |  |  |
|  |  |  |  |  |  |
| **DV: Intrusive Distraction** | | |  |  |  |
|  | Estimate | Std. Error | Df | t-value | p-value |
| (Intercept) | -0.01 | 0.08 | 118.8 | -0.08 | .934 |
| gradient1 | 0.02 | 0.03 | 2045 | 0.68 | .500 |
| gradient2 | 0.05 | 0.04 | 2045 | 1.34 | .412 |
| gradient3 | -0.04 | 0.03 | 2043 | -1.16 | .412 |
| gradient4 | 0.12 | 0.05 | 2048 | 2.17 | .152 |
| gradient5 | -0.06 | 0.07 | 2046 | -0.89 | .470 |
|  |  |  |  |  |  |
|  |  |  |  |  |  |
|  |  |  |  |  |  |
| **DV: Verbal Detail** | |  |  |  |  |
|  | Estimate | Std. Error | Df | t-value | p-value |
| (Intercept) | 0.01 | 0.09 | 119 | 0.05 | .963 |
| gradient1 | -0.01 | 0.03 | 2043 | -0.27 | .787 |
| gradient2 | -0.04 | 0.04 | 2043 | -1.03 | .382 |
| gradient3 | 0.03 | 0.03 | 2042 | 1.03 | .382 |
| gradient4 | 0.09 | 0.05 | 2046 | 1.71 | .217 |
| gradient5 | -0.15 | 0.07 | 2045 | -2.06 | .199 |
|  |  |  |  |  |  |
|  |  |  |  |  |  |
| **DV: Sensory Engagement** | | |  |  |  |
|  | Estimate | Std. Error | Df | t-value | p-value |
| (Intercept) | 0.01 | 0.07 | 119.15 | 0.16 | .870 |
| gradient1 | -0.09 | 0.03 | 2046 | -3.26 | .006 |
| gradient2 | 0.03 | 0.03 | 2046 | 0.78 | .432 |
| gradient3 | 0.03 | 0.03 | 2044 | 1.17 | .404 |
| gradient4 | 0.07 | 0.05 | 2049 | 1.55 | .204 |
| gradient5 | 0.05 | 0.06 | 2048 | 0.86 | .432 |
